# Exogenous Application of 24-Epibrassinolide Confers Saline Stress and Improves Photosynthetic Capacity, Antioxidant Defense, Mineral Uptake, and Yield in Maize

**DOI:** 10.3390/plants12203559

**Published:** 2023-10-13

**Authors:** Mahmoud F. Seleiman, Awais Ahmad, ElKamil Tola, Bushra Ahmed Alhammad, Khalid F. Almutairi, Rangaswamy Madugundu, Khalid A. Al-Gaadi

**Affiliations:** 1 Plant Production Department, College of Food and Agriculture Sciences, King Saud University, P.O. Box 2460, Riyadh 11451, Saudi Arabia; 2 Department of Crop Sciences, Faculty of Agriculture, Menoufia University, Shibin El-Kom 32514, Egypt; 3 Precision Agriculture Research Chair, Deanship of Scientific Research, King Saud University, Riyadh 11451, Saudi Arabia; 4 Biology Department, College of Science and Humanity Studies, Prince Sattam Bin Abdulaziz University, Al Kharj Box 292, Riyadh 11942, Saudi Arabia; 5 Department of Agricultural Engineering, College of Food and Agriculture Sciences, King Saud University, Riyadh 11451, Saudi Arabia

**Keywords:** 24-epibrassinolide, exogenous EBL, saline stress, mitigator, maize

## Abstract

Salinity is one of the major environmental stresses threatening crop production, the natural ecosystem, global food security, and the socioeconomic health of humans. Thus, the development of eco-friendly strategies to mitigate saline stress and/or enhance crop tolerance is an important issue worldwide. Therefore, this study was conducted during the summer of 2022 to investigate the potential of 24-Epibrassinolide (EBL) for mitigating saline stress and improving photosynthetic capacity, antioxidant defense systems, mineral uptake, and yield in maize (*Zea mays* L.) grown under a controlled hydroponic system. Three saline stress levels—S1 (control/no added NaCl), S2 (60 mM NaCl), and S3 (120 mM NaCl)—were continuously applied with nutrient solution, whereas exogenous EBL (i.e., control, 0.1 µM and 0.2 µM) was applied as exogenous application three times (i.e., 40, 55, 70 days after sowing). The experiment was designed as a split-plot in a randomized complete block design (RCBD) in which saline stress was the main factor and EBL treatment was the sub-factor. Results showed that saline stress significantly affected plant growth, physiological performance, biochemistry, antioxidant activity, and yield attributes. However, the exogenous application of EBL at 0.2 µM significantly mitigated the salt stress and thus improved plant performance even under 120 mM NaCl saline stress. For instance, as compared to untreated plants (control), 0.2 µM EBL application improved plant height (+18%), biomass (+19%), SPAD (+32%), Fv/Fm (+28%), rate of photosynthesis (+11%), carboxylation efficiency (+6%), superoxide dismutase (SOD +14%), catalase (CAT +18%), ascorbate peroxidase (APX +20%), K^+^ (+24%), 100-grain weight (+11%), and grain yield (+47%) of maize grown under salt stress. Additionally, it resulted in a 23% reduction in Na^+^ accumulation in leaves and a 25% reduction in for Na^+^/K^+^ ratio under saline stress as compared to control. Furthermore, the Pearson’s correlation and principal component analysis (PCA) highlighted the significance of exogenous EBL as saline stress mitigator in maize. Overall, our results indicated the protective effects of EBL application to the alleviation of saline stress in crop plants. However, further exploration of its mechanism of action and crop-specific response is suggested prior to commercial use in agriculture.

## 1. Introduction

Among the major abiotic factors that threaten sustainable agricultural production and global food security, soil salinity is becoming a serious global problem [1,2]. In addition to the natural process of soil salinization, anthropogenic activities such as inappropriate soil management, low quality irrigation water, and overfertilization of crops further amplify the issue [3]. Currently, over a billion hectares of land have already been affected by salinity, further spreading at the rate of over two megahectares per annum [4,5,6]. Nearly 20% of total farmland and 33% of the irrigated land are suffering through considerable salinity and much more is susceptible [7]. More obviously, semiarid and arid regions are more vulnerable to soil salinity due to limited rainfall, changing climate patterns, global warming, and high evapotranspiration coupled with drought stress [8,9]. The presence of saline stress causes additional pressure on crop plants in order to manage stress, such as biosynthesis of antioxidants, osmoregulation, active mineral uptake, protein synthesis, and alteration of physicochemical mechanisms [10]. If left unattended, it could affect 50% of agricultural land by 2050, which—in addition to serious food insecurity—may affect ecological well-being, availability of natural resources, and socioeconomic health of humans in the long run [2,9].

The accumulation of soluble salts in soil, especially in the root zone, is generally referred to as “soil salinization”. Soil salinity negatively affects the soil’s biochemical nature and biological activity, which consequently lowers the efficacy of sustainable crop production [11]. Exposure to saline stress significantly affects all growth and development stages of plants from germination to senescence [2,8,9]. Overaccumulation of soluble salts in soil solution leads to physiological drought, ionic toxicity, nutrients depletion, and subsequent oxidative stress in plants [8,11,12]. At the cellular level, saline stress promotes the formation of reactive oxygen species (ROS) which results in damaged cell structures through the denaturation of biomolecules and physiological dysfunctionality [13,14,15]. In addition to impaired cell cycle and mitosis, saline stress lowers cell turgor pressure and disrupts chemiosmosis and ultimately cell elongation and hence plant growth [16,17]. Furthermore, damaged photosynthetic machinery and stomatal closure can lead to a reduction in net CO_2_ assimilation and carbon partitioning and hence the economic yield [9,18]. Ionic toxicity as a result of Na^+^ and Cl^−^ accumulation in cell compartments interrupts cellular metabolism, enzymatic activity, and biochemical pathways which eventually result in cell death [19,20]. Moreover, excessive cytoplasmic Na^+^ interferes with the uptake and transportation of cationic nutrients, such as K^+^, Zn^2+^, Ca^2+^, and Mg^2+^, and nitrogen and phosphorus molecular ions [21,22,23]. Conventional soil salinity management approaches, such as scrapping, flushing, supplementation, leaching, and ploughing, often fail to stop/ameliorate saline stress [7]. Therefore, modern technologies aimed at enhancing saline stress tolerance in plants have been investigated more recently. The exogenous use of various chemicals, including osmoprotectants, nanofertilizers, micronutrients, antioxidants, and plant growth regulators, has been reported as a potentially viable and economical approach to mitigating abiotic stresses in plants including saline stress. However, the mechanism of their action, optimization of effective dose, and efficacy of their commercial use are still unclear and need further exploration.

Brassinosteroids (BRs) are phytohormones in nature and are ubiquitous in the plant kingdom. They are generally referred to as plant growth regulators or hormones with pleiotropic effects which can affect a wide spectrum of developmental processes in plants, such as germination, rhizogenesis, growth, flowering, and fruit setting. Furthermore, BRs can confer resistance to plants against biotic and abiotic stresses [24,25,26]. 24-epibrassinolide (EBL) is an active byproduct of brassinolide biosynthesis in plants and has been successfully produced chemically [27]. Under normal conditions, EBL stimulates various plant metabolic processes including ATP synthesis, ROS metabolism, biosynthesis of nucleic acid, and CO_2_ assimilation. Moreover, it regulates various key enzymes involved in photosynthesis, cell cycle, and homeostatic response in plants [28]. Exogenous application of EBL in plants showed potential to mitigate abiotic stresses, such as drought [29], heat [27], heavy metals [30], and salinity [31]. Even though EBL’s mechanism of action in plants is not fully understood yet, a number of international organizations have reported it as environmentally safe, eco-friendly and exogenously applicable on leafy plants [32]. Considering the commercial application of EBL in agriculture as a potential strategy to alleviate abiotic stresses in plants by enhancing their tolerability is still relatively new and unexplored. Furthermore, crop-specific response to exogenous application of EBL and optimum dose needs to be further evaluated.

Maize (*Zea mays* L.), after wheat and rice, is the third major important crop meeting global requirements of food, feed, and industries [33]. As a result of its wider genetic variability and large adaptability, its area of production has increased over 70% since the green revolution has [34]. Recently, it is being cultivated in more than 155 countries under nearly 170 climatic conditions with an annual average production of 1.2 billion tons in 2021, it has surpassed both rice and wheat [35,36,37]. However, the prevailing abiotic stresses are challenging its sustainable production and thus global food security. Despite all the advances made in maize development, it is still generally referred to as “salt sensitive”.

Therefore, using the recent advances made in agricultural technologies and understanding of the mechanism of saline stress in plants, the development of new strategies to mitigate the deleterious effects of saline stress in maize is of vital importance for sustainable food production. Thus, the present study was designed to thoroughly investigate the effect of exogenous application of EBL (i.e., control, 0.1 µM and 0.2 µM) on maize grown under controlled saline stress (control = S1; 60 mM NaCl = S2; and 120 mM NaCl = S3).

## 2. Materials and Methods

### 2.1. Experimental Design Treatments and Growth Conditions 

#### 2.1.1. Establishing the Nursery

Prior to the sowing process, seeds of maize (*Zea mays* L.; Hybrid-310) were treated with sodium hypochlorite (NaOCl) solution (1.0% *w*/*v*) for 15 min and washed with tap water thoroughly to remove NaOCl residue. Then, seeds were sown at a depth of 1.0 cm in peat moss filled in germination trays made up of plastic. Each tray consists of 96 cells, each of 45 cm^3^ volume (5 cm × 3 cm × 3 cm). Initially, three seeds were placed in each cell, and after one week of germination, one seedling per cell was maintained through thinning. After watering at field capacity, germination trays were placed in a semi-controlled glasshouse at the College of Food and Agriculture Sciences, King Saud University, Saudi Arabia. The glasshouse was equipped with an artificial cooling system to maintain temperature 28 ± 1 °C for day and 23 ± 1 °C at night along with relative humidity ranging between 52–60%. The light was set at 14 h of light and 10 h of dark per day. The nursery was irrigated after each three-day regular period for three weeks after sowing.

#### 2.1.2. Transplantation and Growth Conditions

Seedlings 21 days of age were shifted to an environmentally controlled glass made greenhouse facility at Educational Farm, College of Food and Agricultural Sciences, King Saud University, Kingdom of Saudi Arabia. The greenhouse was equipped with an auto-control semi-closed irrigation system with hydroponic arrangement. Twelve rows, each of 28 m long steel made of strands raised 30 cm from the floor, were facilitated with an independent irrigation system. The seedlings were transplanted in agricultural perlite (average diameter of 10 mm) packed in polythene bags (100 cm × 25 cm × 25 cm) mounted on steel strands. The plant-to-plant distance was maintained at 20 cm. Thus, four seedlings were planted per bag. Plants were irrigated for 2 min twice a day for the first three weeks and then gradually increased to four irrigations near to physiological maturity. The greenhouse environment was maintained at 13 h of light and 11 h dark, 60–65% relative humidity and 30 ± 1 °C and 23 ± 1 °C day and night temperatures, respectively.

#### 2.1.3. Treatments and Experimental Design

Three saline stress levels as; S1 (control, no added NaCl), S2 (60 mM NaCl), and S3 (120 mM NaCl) were applied with plant nutrient solution as reported by Basit et al. [38]. The pH and electric conductivity (EC) of nutrient solution were adjusted as 5.5 pH and 1.1 dS m^−1^, respectively. The plants were treated with foliar application of 24-Epibrassinolide (EBL) (i.e., control, 0.1 µM and 0.2 µM) sprayed three times at 40, 55, and 70 days after sowing (DAS). The experiment was designed as a split-plot in a randomized complete block design (RCBD) in which saline stress was the main factor and EBL treatment was the sub-factor. Each experimental unit (sub-factor) consisted of 3 m rows, and the experiment was replicated thrice.

### 2.2. Measurements

#### 2.2.1. Evaluation of Plant Growth Parameters and Mineral Ions

Maize plant growth performance was evaluated at 90 DAS. The plant height (cm) was recorded for five plants per treatment using a meterstick. Stem girth (mm) was measured with the help of a vernier caliper, with which the stem diameter was taken from three different directions at a height of 5 cm from the first adventitious root. In order to measure fresh and dry weight per plant (g), three plants were harvested and weighed immediately (FW). Using the same plant samples, leaf area per plant (cm^2^) was measured with the help of a Li-COR portable leaf area meter, LI-3000C (LI-COR, Lincoln, NE, USA). The plants were then oven dried at 70 ± 1 °C for 72 h and dry weight was recorded (DW). The specific leaf weight (SLW) (mg/cm^2^) was computed as the ratio of leaf dry weight to leaf area. Disc-shaped (2 cm in diameter) leaf samples were taken from freshly harvested plants and weighed immediately, followed by 12 h immersion in distilled water to obtain turgor weight. The leaf samples were then dried in an electric oven at 70 ± 1 °C for 48 h, and dry weight was recorded. The leaf relative water contents (RWC) were calculated using the following equation.

(1) $$RWC=\frac{Turgor\;weight-Fresh\;weight\;\;}{Turgor\;weight-Oven\;dry\;weight\;}\times \;100$$

Moreover, the leaf growth rate (LGR) and plant growth rate (PGR) were computed as plant growth indicators. At the 6th week of sowing, plants were tagged on the 6th leaf from bottom, and leaf area was measured for eight consecutive weeks (42, 49, 56, 63, 70, 77, 84 and 91 DAS) using a portable leaf area meter (LI-3000C, LI-COR, Lincoln, NE, USA). The leaf growth rate was computed as a per day increase in leaf area (cm^2^/day). Similarly, plant growth rate was computed as change in plant height per day (cm/day) for eight weeks starting from 6th week after sowing. In order to determine Na^+^ and K^+^ contents (g/kg), dry leaf samples (500 mg) were powered and digested as described by Wolf [39]. The extract was then analyzed using a flame photometer (Corning 400, Sherwood Scientific Ltd., Cambridge, UK).

#### 2.2.2. Photosynthetic and Physiological Traits Assessment

The maximum quantum yield of photosystem II (Fv/Fm) was measured using a portable Handy PEA+ (Hansatech Instruments Ltd., Norfolk, UK), for which fully expanded maize leaves were clipped for 30 min for dark adaptation. Leaf green index (LGI) was recorded as a soil plant analysis development (SPAD) reading using a SPAD 502 Plus (Spectrum Technologies, Bridgend, UK). Both Fv/Fm and LGI were measured 90 DAS. The gas exchange and photosynthetic traits such as rate of photosynthesis (Pn), transpiration rate (Tr), intercellular CO_2_ concentration (Ci) and stomatal conductance (Sc) were measured from fully expanded leaves using portable LI-6400XT photosynthetic system (LI-COR, Li-COR, Lincoln, NE, USA) 90 DAS. All measurements were taken between 9:00–11:00 a.m. from five leaves per treatment. During the measurements the gas chamber maintained at 25 ± 1 °C temperature, 60–70% relative humidity, 800 m^− 2^ s^− 1^ photosynthetic photon flux density (PPFD) and 400 μmol·mol^− 1^ CO_2_ concentration. The data obtained were further processed to compute carboxylation efficiency (CE) and intrinsic water use efficiency (iWUE) as Pn/Ci and Pn/Sc, respectively. 

#### 2.2.3. Antioxidant Enzymatic Activity, Proline and Total Phenolic Contents

After eleven weeks of sowing 0.5 g of fresh leaf samples were taken from five plants per treatment and processed immediately for antioxidant enzyme activity. In liquid nitrogen, the samples were ground to powder and homogenized with 50 mM phosphate buffer solution (7.0 pH). The mixture was centrifuged using a Benchtop Centrifuge-5810R (Eppendorf, Hamburg, Germany) for 2.0 min at 15,000 rpm and 4 °C [40]. The collected supernatant was then used for subsequent enzymatic activity assay. Following the method described by Jiang and Zhang [40], H_2_O_2_ consumption at 240 nm for 3 min was taken for catalase (CAT) activity. Whereas, for ascorbate peroxidase (APX) activity, ascorbate consumption was analyzed at 270 for 60 s as described by Zhu et al. [41]. To determine superoxide dismutase (SOD) activity, the Kong et al. [42] method was used; 50% inhibition of nitro blue tetrazolium (NBT) was taken as one unit of SOD activity. 

Similarly, at the end of the 11th week after sowing, total phenolic contents were also determined. For this, 250 mg of fresh leaf sample was taken from five plants per treatment. The samples were ground to powder in liquid nitrogen, and phenolic contents were extracted using 80% ethanol at 37 °C [43]. The extract was then cooled to 4 °C and centrifuged at 3500 rpm after homogenization with methanol. The total phenolic contents (mg GAE/g FM) were estimated using the Folin–Ciocalteu colorimetric method [44]. In order to determine proline contents, 5.0 g samples of fresh leaves were homogenized with 10 mL of 3% (*v*/*v*) sulfosalicylic acid followed by 10 min centrifugation at 5000 rpm using Benchtop Centrifuge-5810R (Eppendorf, Hamburg, Germany). The 2 mL supernatant obtained was then mixed with 2 mL of glacial acetic acid and 2 mL of ninhydrin and incubated in a hot water bath at 94–100 °C followed by ice shock. Following the method defined by Bates et al. [45], the proline contents (mg/g FW) in samples were estimated using standard curve of known proline concentration at 520 nm with the help of UV–VIS spectrophotometer (SHIMADZU, Kyoto, Japan, UV1800). 

#### 2.2.4. Yield and Yield Attributes

The maize plants were harvested 17 weeks after sowing (114 DAS), when all leaves became fully dry. The ears per plant were sun dried separately for 3 days in open air to a constant weight. The maize yield and yield attributes—i.e., number of grains per ear, 100-grain weight (g), grain weight per ear (g), and grain yield per plant (g)—were measured. 

### 2.3. Statitical Analysis

The data collected were subjected to PASW statistics 21.0 (IBM Inc., Chicago, IL, USA) for analysis of variance (ANOVA). Least significant difference (LSD) was used to compare treatment means. The standard error (±SE) was obtained and presented in Figures to show the differences between different treatments. All parameters were statistically processed individually, and readings taken at different times were processed independently. The Pearson’s correlation coefficient was calculated using XLSTAT statistical package (Version 2018, Excel Add-ins soft SARL, New York, NY, USA). The mean data of three replications were subjected to Euclidean distance (version 3.11), and principal component analysis (PCA) was performed.

## 3. Results

The analysis of variance (ANOVA; Table 1) shows that the 28 studied parameters of maize plants were significantly (*p* ≤ 0.05) affected by saline stress. Moreover, the exogenous application of EBL also significantly (*p* ≤ 0.05) affected the study parameters except intrinsic water use efficiency (iWUE). The interactive relationship between saline stress and exogenous application of EBL on maize was significant (*p* ≤ 0.05) for most of the parameters expect; plant height, biomass, PGR, intercellular CO_2_ concentration (Ci), iWUE, number of grains per ear, and grain weight per ear, where salinity × EBL was noted to be statistically nonsignificant.

The saline stress impaired plant growth in maize significantly. However, the magnitude of deleterious effects of saline stress increased with increasing intensity of saline stress from S1 to S2 and S3, and S3 resulted in the lowest values for all plant growth attributes except SLW. As compared to control (S1), the saline stress at 120 mM NaCl (S3) reduced plant height, stem girth, biomass, leaf area per plant, and leaf RWC by −18%, −12%, −24%, −33%, and −20%, respectively, whereas SLW increased by 16% (Table 2). The exogenous application of EBL however alleviated the saline stress mediated decline in plant growth attributes of maize where 0.2 µM EBL stood significantly higher than all other treatments. The EBL at 0.2 µM improved plant height (+18%), stem girth (+16%), biomass (+19%), leaf area (+35), and leaf RWC (+5%) as compared to control but lowered SLW by −8% (Table 2). The interactive effect of S1 and 0.2 µM EBL resulted in maximum stem girth (19.67 mm) and leaf area per plant (7514 cm^2^) and minimum SLW (49.13 g/m^2^). Oppositely, the combined effect of saline stress control (S1) and EBL control produced lowest stem girth (15.07 mm), leaf area per plant (3353 cm^2^), and leaf RWC (63.27%) and highest SLW (62.67 g/m^2^) (Table 2).

**Figure 1:**
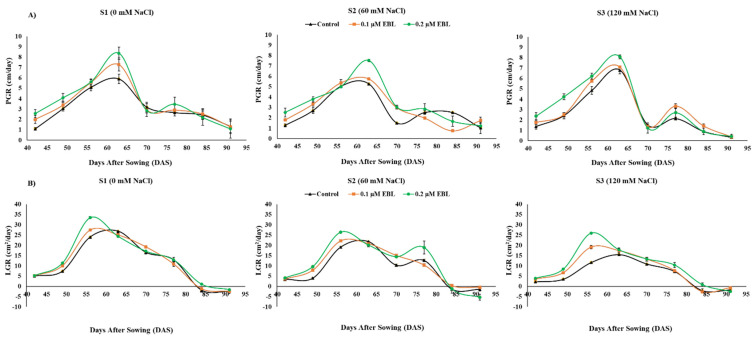
Effect of exogenous application of 24-Epibrassinolide (EBL) on (**A**) plant growth rate (PGR) and (**B**) leaf growth rate (LGR) of maize grown under saline stress. Bars are standard error (±SE).

The plant growth rate (PGR) and leaf growth rate (LGR) were continuously measured for eight weeks at seven-day intervals, starting from 42 DAS. Results revealed that the initial PGR (Figure 1A) between 42 to 63 DAS stood higher than the second growth half from 63 DAS to 91 DAS for all saline stress intensities. Moreover, on average, the EBL application at 0.2 µM improved PGR as compared to EBL control, and significant differences were seen at 42, 49, 63, and 77 DAS under all saline stress conditions. Nearly similar findings were recorded for LGR (Figure 1B), for which between 42 and 56 DAS, the LGR continuously increased under all combinations of saline stress and EBL applications and reversed later and became negative after 84 DAS. Once again, foliar application of 0.2 µM EBL resulted in higher average LGR compared to control and 0.1 µM EBL. The most significant differences were recorded at 49, 56, 70, 77, and 84 DAS. In general, 120 mM NaCl saline stress lowered growth rate as compared to control (S1), whereas the exogenous application of EBL ameliorated salinity induced decline both in PGR and LGR (Figure 1). 

**Figure 2:**
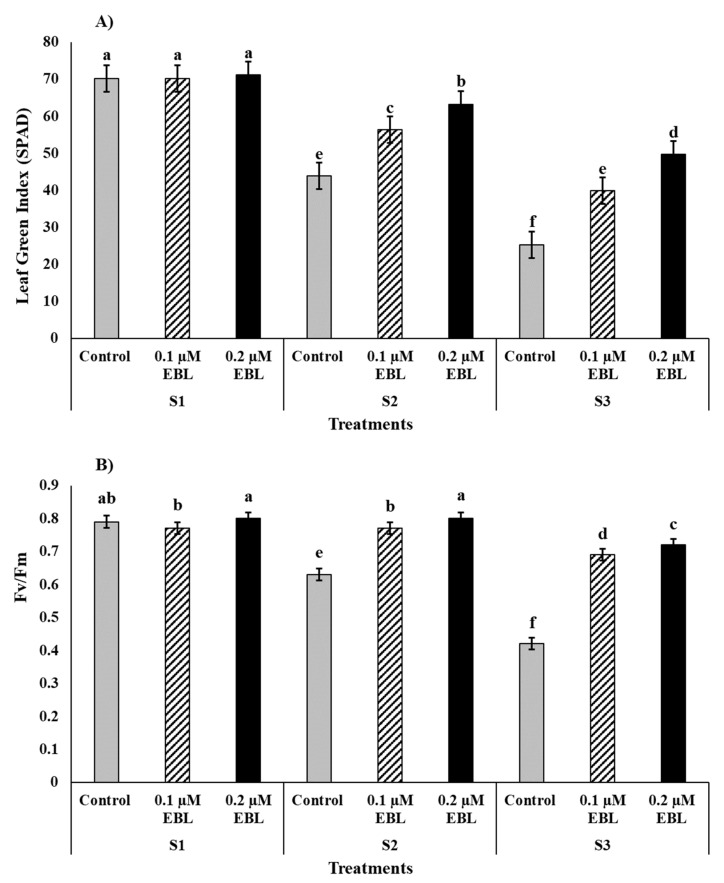
Effect of exogenous application of 24-Epibrassinolide (EBL) on (**A**) leaf green index (LGI) (SPAD) and (**B**) maximum photochemical efficiency of PSII (Fv/Fm) of maize grown under saline stress. Bars are standard error (±SE). Small letters show the significance differences between the interaction effects of S × EBL.

Saline stress in maize significantly affected the leaf green index (LGI) (SPAD-reading) and maximum photochemical efficiency (Fv/Fm) thus, saline stress at 120 mM NaCl (S3) as compared to control decreased LGI and Fv/Fm by −43% and −23% respectively (Figure 2). However, the exogenous application mitigated the saline stress mediated decline in LGI (Figure 2A) and Fv/Fm (Figure 2B). The EBL at 0.2 µM and improved both LGI (+32) and Fv/Fm (+28%) compared to EBL control. The combined treatments of S1 with EBL control, 0.1 µM, and 0.2 µM were found statistically equal, and the highest were the 70.18, 70.18, and 71.08 SPAD readings for LGI. The lowest values (25.2) of LGI were recorded for combinations of S3 (120 mM NaCl) with EBL control. Similarly, for Fv/Fm, the combinations of S1 and EBL—both as control—and 0.1 µM were noted as the highest but equal to 0.80 and 0.79, respectively. However, the lowest Fv/Fm (0.42) was again found for combined treatment of S3 and EBL control.

**Figure 3:**
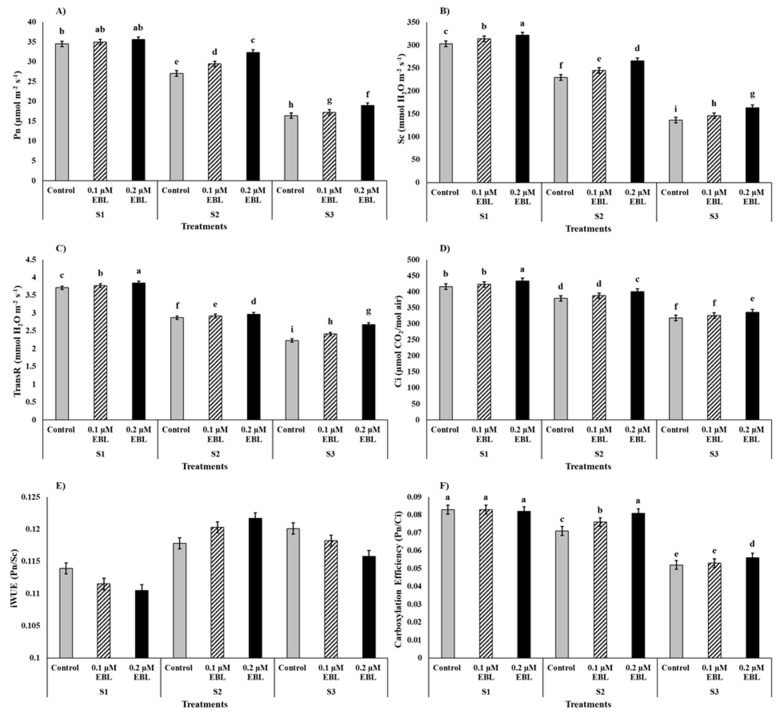
Effect of exogenous application of 24-Epibrassinolide (EBL) on (**A**) rate of photosynthesis (Pn), (**B**) stomatal conductance (Sc), (**C**) transpiration rate (TransR), (**D**) intercellular CO_2_ concentration (Ci), (**E**) intrinsic water use efficiency (iWUE), and (**F**) carboxylation efficiency (CE) of maize grown under saline stress. Bars are standard error (±SE). Small letters show the significance differences between the interaction effects of S × EBL.

The saline stress significantly hampered photosynthesis and related physiological attributes of maize plants (Figure 3). As compared to saline control (S1), the 120 mM NaCl (S3) saline stress decreased the rate of photosynthesis (Pn), stomatal conductance (Sc), transpiration rate (TransR), intercellular CO_2_ concentration (Ci), and carboxylation efficiency (CE) by −50%, −52%, −35%, −23%, and −35%, respectively, whereas it increased intrinsic water use efficiency (iWUE) by 5%. However, the foliar application of EBL improved photosynthetic performance for maize plants by ameliorating the saline-stress-mediated decline of the aforementioned parameters (Figure 3). The EBL application at 0.2 µM as compared to control enhanced Pn, Sc, TransR, Ci and CE by +11%, +12%, +8%, +5%, and +6% respectively. Additionally, the interactive effect of salinity and EBL application was also significant for all above mentioned parameters except iWUE. The combination of S3 and EBL control resulted in the lowest Pn (Figure 3A), Sc (Figure 3B), and TransR (Figure 3C). The combinations of S3 with EBL control and 0.1 µM EBL showed statically similar but lower values for Ci (Figure 3D) and CE (Figure 3F) compared to all other combinations of treatments. On the other hand, maximums of Sc (Figure 3B), TransR (Figure 3C), and Ci (Figure 3D) were recorded for the combination of saline stress control (S1) and 0.2 µM EBL. However, the combinations of S1 both with 0.1 µM EBL and 0.2 µM EBL resulted in statistically at par but maximum values of Pn (Figure 3A) compared to all other combinations of treatments.

Maize plants under saline stress significantly increased the biosynthesis of proline and antioxidant enzymatic activity (i.e., SOD, CAT, APX), where S3 (120 mM NaCl) compared to control (S1) resulted in +548%, +80%, +106%, and +86% higher proline content and SOD, CAT, and APX activities, respectively (Table 3). Interestingly, the exogenous application of EBL showed synergistic effect on salinity and further increased the antioxidant enzymatic activity. The SOD, CAT, and APX activities under 0.2 µM EBL compared to untreated plants were increased by +14%, +18%, and +20%, respectively (Table 3). Moreover, 0.2 µM EBL application decreased the saline stress mediated over production of proline by −54% as compared to control. Inconsistent to general trends, the maximum total phenolic contents were found for S2 and 0.2 µM EBL as +24% and +14% higher than their respective controls (Table 3). The combined treatment of maximum saline stress S3 (120 mM NaCl) and EBL (0.2 µM) produced highest antioxidant enzymatic activity in maize leaves as; 192.00 (U/gFW) SOD, 67.33 (U/gFW/min) CAT and 39.67 (umol/gFW/min) APX. The highest proline contents (3.83 mg/gFW) were measured for the combined effect of S3 and EBL control, whereas the highest total phenolic contents (60.12 mgGAE/gFM) were found for the interactive effect of S2 and 0.2 µM EBL (Table 3).

**Figure 4:**
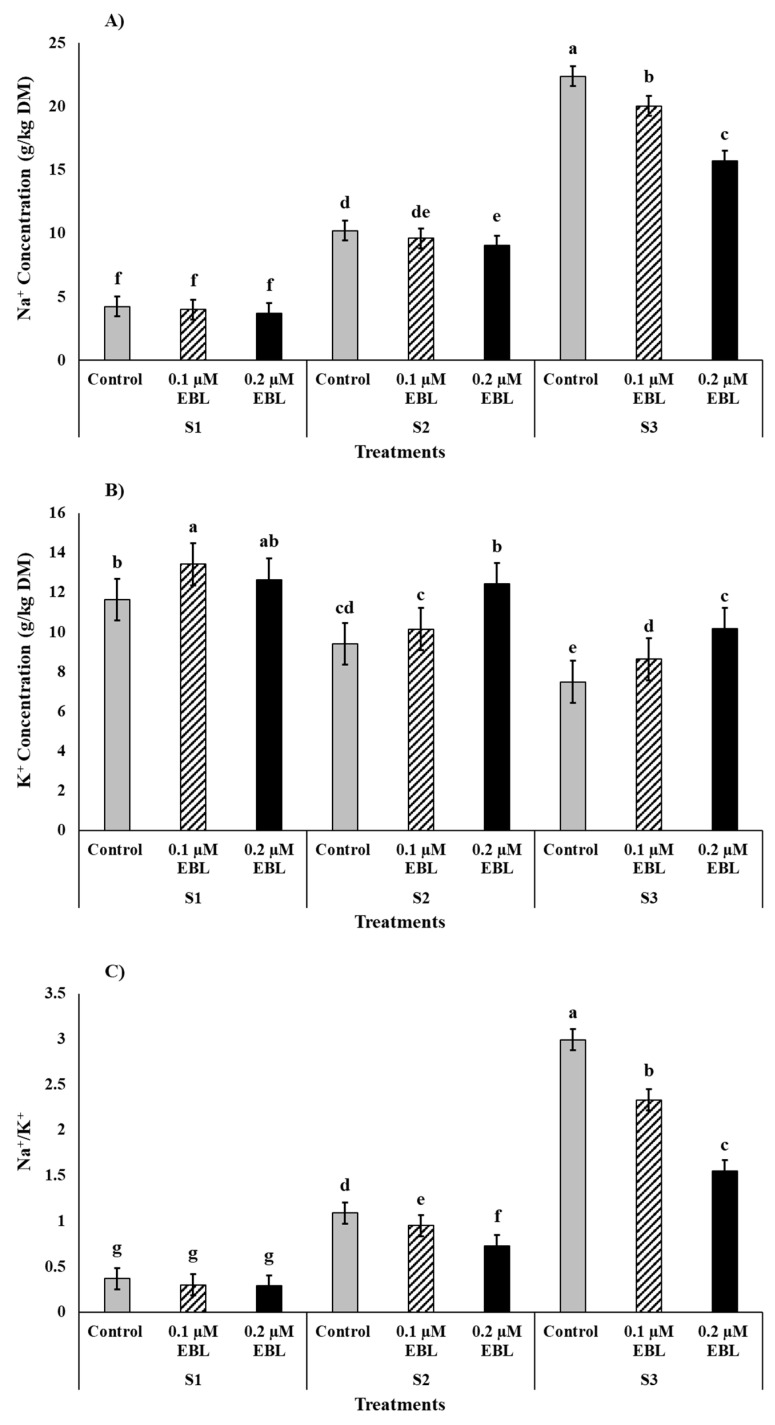
Effect of exogenous application of 24-Epibrassinolide (EBL) on (**A**) Na^+^ concentration, (**B**) K^+^ concentration, and (**C**) Na^+^/K^+^ of maize grown under saline stress. S1 = 0 mM NaCl, S2 = 60 mM NaCl, S3 = 120 mM NaCl. Bars are standard error (±SE). Small letters show the significance differences between the interaction effects of S × EBL.

The Na^+^ concentration in maize plant leaves increased significantly with increasing saline stress intensity, such as by 3.99, 9.63, and 19.63 g/kg for DW samples S1, S2, and S3, respectively. Thus, 120 mM NaCl (S3) resulted in 385% higher Na^+^ compared to control (S1) (Figure 4A). Contrary to Na^+^, the K^+^ concentration decreased by −30% for S3 compared to saline control S1 (Figure 4B). Thus, the higher Na^+^ and lower K^+^ resulted in +615% higher Na^+^/K^+^ for maximum higher saline stress (S3) compared to S1 (Figure 4C). However, the exogenous application of EBL significantly ameliorated the ionic imbalance; 0.2 µM EBL lowered Na^+^ concentrations by −23% and Na^+^/K^+^ by −25%, whereas it improved K^+^ by +24% as compared to EBL control (Figure 4). The interactive effect of 120 mM NaCl (S3) and EBL control resulted in maximum Na^+^ concentration (22.37 g/kg DW) and Na^+^/K^+^ (2.99), whereas the maximum K^+^ contents were measured for combined treatments of saline control (S1) with 0.1 µM and 0.2 µM EBL as 13.41 and 12.64 g/kg DW, respectively (Figure 4).

The deleterious effects of saline stress significantly decreased grain yield and yield-related attributes for 120 mM NaCl saline stress (S3) compared to the control and lowered the number of grains per ear, grain weight per ear, 100-grain weight, and grain yield per plant by −53%, −69%, −10%, and −73%, respectively (Table 4). However, the exogenous application of EBL alleviated the deleterious effects of saline stress on maize yield and yield attributes. The EBL at 0.2 µM compared to control (no EBL) improved the number of grains per ear (+31%), grain weight per ear (+43%), 100-grain weight (+11%), and grain yield per plant (+47%). The interactive effect of saline stress and EBL was nonsignificant for number of grains per ear and grain weight per ear. However, the maximum 100-grain weight (28.53 g) and grain yield per plant (203.97 g) were recorded for the combined effect of S1 and 0.2 µM EBL. Moreover, the combination of S3 (120 mM NaCl) and EBL control resulted in the lowest 100-grain weight (16.63 g) and grain yield per plant (27.07 g) (Table 4).

**Figure 5:**
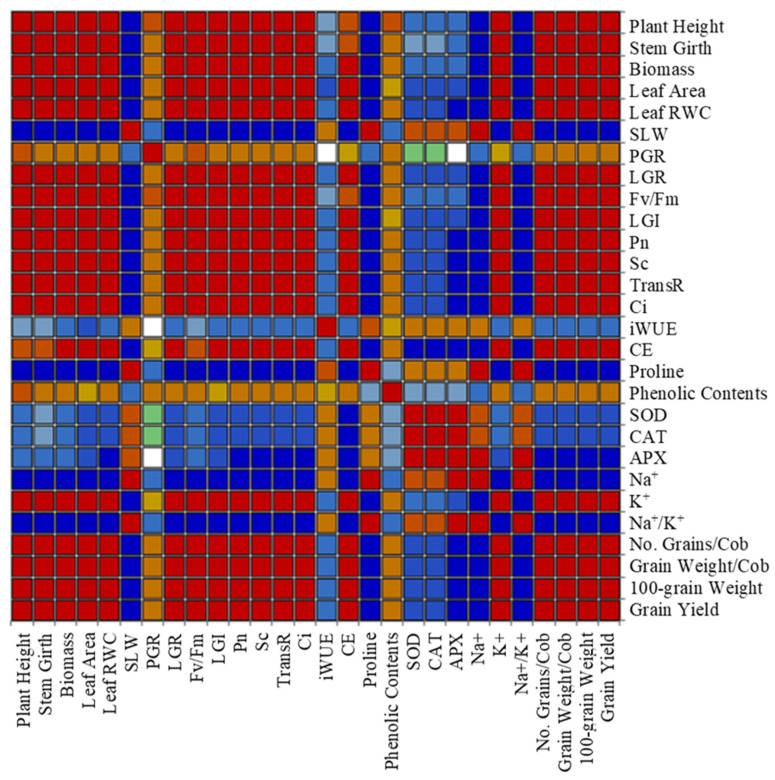
Pearson’s correlation matrix of various growth, physiological, biochemical, and yield attributes of maize under saline stress and EBL exogenous application. Red color codes a strong positive correlation, and blue represents a strong negative correlation. For abbreviations, see previous tables and figures.

The Pearson’s correlation was conducted to determine the effect of exogenous application of EBL and saline stress on maize. In general, most of the studied parameters showed a strong positive relationship. However, SLW, proline contents, Na^+^ concentration, Na^+^/K^+^ ratio, and antioxidant enzymatic activity (i.e., SOD, CAT, and APX) showed negative correlations with growth, physiological, and yield parameters. The 100-grain weight and grain yield per plant were found to be significantly/strongly correlated with growth and physiological attributes in addition to K^+^ contents, which were affected by EBL treatment and saline stress (Figure 5). 

**Figure 6:**
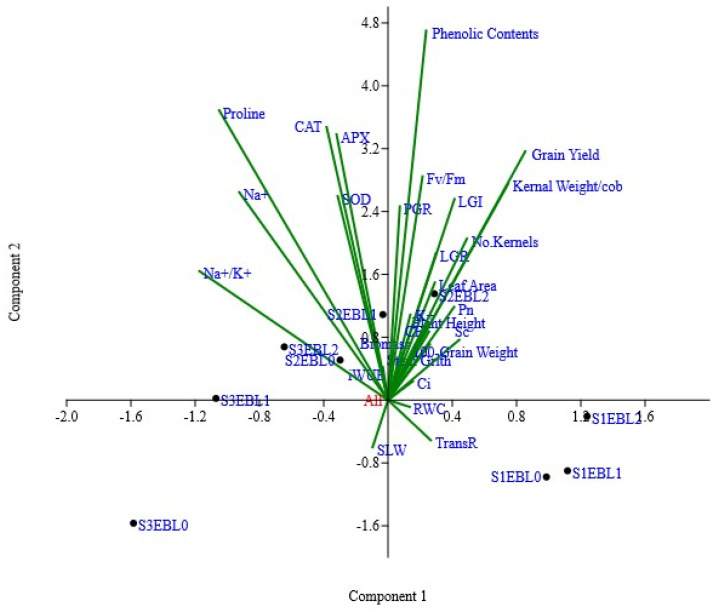
Biplot analysis (PC1 and PC2) of combined data for exogenous application of EBL and saline stress for all studied parameters of maize plants. For abbreviations, see previous tables and figures.

The PCA was performed to examine the combined effect of EBL treatment and saline stress on maize plant performance. The results revealed that the first two principal components (PCs) accounted for a total variance of 95.34%. The biplot analysis of PC1 and PC2 highlighted the significant characteristics that contributed to the observed diversity within the parameters. These characters included PGR, Fv/Fm, LGI, LGR, leaf area, Pn, plant height, 100-grain weight, stem girth, grain yield per plant, and grain weight per ear, which were predominantly present in the first quadrate of PC1. Additionally, RWC and TransR were present in the second quadrate of PC1. Similarly, in PC2, the most influential characters were CAT, APX, SOD, proline, Na^+^, iWUE, and Na^+^/K^+^. These characters demonstrated remarkable performance under high salinity stress and EBL application (Figure 6). Most of the control treatments with EBL and low-level salinity stress (S1, S2) were present in the PC1. However, the EBL and high-level salinity stress (S3) scattered in the PC2.

## 4. Discussion

### 4.1. Growth and Morphological Attributes 

Saline stress has been recurrently reported for plant growth impairment in agronomic crops [2,10,17]. Maize is generally considered to be sensitive to moderately sensitive to saline stress and therefore suffers a significant decline in growth under salinity stress [46]. In our study, maize growth attributes were significantly reduced by 120 mM NaCl (S3) saline stress (Table 1 and Table 2). Furthermore, the continuous monitoring of PGR and LGR further reflected the saline stress mediated alterations in plant growth (Figure 1). However, the exogenous application of EBL has shown significant alleviation of saline stress and thus resulted in considerably higher growth (Table 1 and Table 2 and Figure 1). The elevated salt accumulation in the root zone destructively influences the plant water relations by creating a negative osmotic pressure and physiological drought [11,12,47]. Therefore, limited availability of water diminished transpiration, nutrient uptake, plant growth, and development [2,9,48]. If saline stress endures, a limited but continuously penetrating of Na^+^ and Cl^−^ accumulates in cellular compartments and results anionic toxicity, which consequently disrupts chemiosmotic balance and metabolism, damages extracellular structures, and may cause cell death [19,20,49]. Furthermore, overaccumulation of ROS leads to oxidative damage of nucleic acid and plasma members, causing electrolyte leakage, which ultimately affects turgor pressure, cell elongation, cell division, and plant growth [15,17,50]. However, EBL application successfully ameliorated saline stress and mediated decline in growth attributes due to its role in regulating cell–water relations, maintenance of turgor pressure, and cell elongation [51,52]. Moreover, EBL has been reported as key hormone required for xylem regeneration, cell division, cell differentiation, and photomorphogenesis, which consequently improves biomass production and thus vegetative growth in plants under abiotic stresses such as salinity [53]. The exogenous application of EBL mitigates ionic toxicity, replenishes leaf RWC, and improves root and shoot lengths, net biomass, and plant growth rates under saline stress [52,54,55]. 

### 4.2. Photosynthetic Activity, Gaseous Exchange, and Physiological Performance

Likewise, photosynthesis and associated physiological trials, such as SPAD-reading, Fv/Fm, Pn, Sc, TransR, Ci, and CE, were significantly reduced by saline stress both at S2 (60 mM NaCl) and S3 (120 mM NaCl) compared to control. However, the exogenous application of EBL as foliar spray effectively mitigated stress-medicated decline in the aforementioned physiological traits (Table 1 and Figure 2 and Figure 3). Comprehensibly, the Na^+^ accumulation in cellar components in response to continuous exposure to saline stress results in swollen chloroplasts and denaturation of the thylakoid membrane [13,56]. Furthermore, saline stress impairs the photosynthetic efficiency of plants by lowering the maximum quantum yield of photosystem II (PS II), reducing non-photochemical quenching (NPQ)—inducing photo-inhibition—and reducing electron transport rate (ETR) due to over-excitation of energy [57,58]. Furthermore, saline stress can hinder enzymatic activity and nutrient uptake and thus the biosynthesis of photosynthetic pigments, which further lowers carboxylation efficiency and net CO_2_ assimilation in stressed plants [9,57]. On the other hand, saline stress can induce a reduction in K^+^, subsequently resulting in the incapability of guard cells and lack of functional osmoregulation, which leads to stomatal closure and lower transpiration rate, thus creating physiological drought at cellular levels impaired photosynthesis and CO_2_ fixation [23,59,60]. Plausibly, the significant increase in physiological and photosynthetic performance of maize plants in response to EBL treatment, which resulted in this study, could be attributed to EBL’s role as a “master hormones” and biochemical crosstalks with other plant growth regulators and hormones [24,53]. Shahzad et al. [53] reported that the exogenous application of EBL preserves a higher rate of photosynthesis via detoxification of Na^+^ hyper-accumulation, nullification of the salinity-induced damage to chloroplast ultrastructures, maintenance of thylakoid membrane integrity, and by enhancing photochemical efficiency of PS II. According to Yue et al. [56], EBL application under 100 mM NaCl stress in *Robinia pseudoacacia* L. significantly improved photosynthetic pigments, stomatal conductance, mesophyll conductance, and reduced intercellular CO_2_ concentration. The improved photosynthesis efficiency in response to EBL application is generally attributed to enhanced NPQ, ETR, Fv/Fm, and sufficient transportation rate along with lower CO_2_ gradient under saline stress conditions [28,53,56].

### 4.3. Antioxidants’ Activity

Saline stress can induce ionic toxicity and physiological drought coupled with oxidative stress thus resulting in overproduction of ROS [61,62]. The hyperaccumulation of ROS in cells induces lipid peroxidation of cellular membranes, inhibits signal transduction, impairs DNA replication and central dogma of protein synthesis, interferes in normal cell functionality, and eventually may lead to cell death [47,63]. The conversion of hydrogen peroxide (H_2_O_2_) and superoxide radial is an integral strategy in plants to withstand environmental stresses. Thus, the enhanced antioxidant enzymatic activity (i.e., SOD, CAT, and APX) is crucial for scavenging ROS in plants under saline stress [63]. Proline acts as a stress indicator in plants and plays an important role in cell membrane stabilization, prevention of protein degradation, and scavenging of free radicals [27]. Our study has depicted significant increases in proline, total phenolic contents, and antioxidant activities of SOD, CAT, and APX in maize plants under saline stress. Interestingly, the exogenous application of EBL further improved antioxidant enzymatic activity and total phenolic contents whereas minimizing the stress-induced production of proline (Table 1 and Table 3). SOD is generally believed to be the first line of defense in antioxidant response mechanisms in plants against ROS and catalyzes superoxide to hydrogen peroxide and molecular oxygen, thus serving under abiotic stress [64]. CAT is predominantly vital for scavenging ROS, and APX possibly regulates cell signaling modulated by ROS in stressed plants [47,63]. Irrefutably, the foliar application of EBL in maize under saline stress facilitated antioxidant defense mechanisms under saline stress and thus contributed to plant growth and physiological performance. Even though the mechanism of its action is yet to be explored, the exogenous application of EBL at 0.2 µM can potentially mitigate saline stress and plants and therefore can be further investigated for better understanding. Exogenous applications of BRs such as EBL under abiotic stresses enhance proline accumulation by promoting biosynthesis and reducing proline degradation and utilization [65]. However, contrary to popular belief, proline contents in maize leaves in the current study were significantly decreased in response to 0.2 µM, which reflects that plants were no longer experiencing the saline stress. Tanveer et al. [31] reported that exogenous application of EBL under saline stress improved plant growth and development by assisting antioxidant defense mechanisms and enhancing antioxidants enzymatic activity. Similar findings were also reported for *Triticum aestivum* [26], *Phaseolus vulgaris* [66], and *Zea mays* [67]. As a saline stress mitigator, exogenous EBL application stimulates enzymatic and non-enzymatic antioxidants by upregulating peroxidase-encoding genes, MAPK3 (mitogen-activated protein kinase 3), MAPK1 (mitogen-activated protein kinase 1), RBOH (respiratory burst oxidase homologue), ATP24a and ATP2 [31,68,69]. 

### 4.4. Mineral Uptake 

Saline stress tolerance management in plants largely depends on the Na^+^ and K^+^ homeostatic mechanisms. Salt stress often causes overaccumulation of Na^+^ ions into cellular compartments and thus lowers K^+^ uptake, remarkably increasing the Na^+^/K^+^ ratio [61,70]. Both Na^+^ and K^+^ share nearly the same hydrated radius, due to which Na^+^ competes with K^+^ at symplast entry sites, resulting in subsequent lower K^+^ concentration in aerial parts [2,61,70]. Moreover, significant down-regulation of genes related to K^+^ inward-rectification channels and K^+^ affinity was reported in *Arabidopsis* root tissues under saline stress [71]. A higher intracellular K^+^ concentration is vital for the efficient maintenance of membrane potentials, optimum activity of several cytosolic enzymes, and biosynthesis of osmoticum [47,70]. The results obtained from maize plants in this study showed a several-times increase in Na^+^ concentration in leaves under 120 mM NaCl stress (S3) compared to control (S1). Likewise, Na^+^ and Na^+^/K^+^ ratio followed the same trend however K^+^ concentration was significantly decreased. The EBL (0.2 µ M) foliar treatment, however, successfully lowered the Na^+^ and Na^+^/K^+^ and improved K^+^ uptake in stressed plants (Figure 4). Several studies have reported the role of exogenous application in mitigating saline stress by decreasing Na^+^ accumulation in cellular compartments and increased K^+^ concentration in aerial plant parts [31,72]. Furthermore, it prevents Na^+^-induced K^+^ leakage from both roots and shoots [73]. 

### 4.5. Yield and Yield Attributes 

Yield losses in agricultural crops due to soil salinization are a prevailing global threat to sustainable food security [9]. However, the yield itself is an aggregative outcome of intricate communications among different plant processes such as growth, development, morphology, physiology, metabolism, and homeostatic and defense mechanisms which become even more important in plants under stress such as salinity [19,74]. Several studies have reported significant decline in physiological performance and photosynthesis efficiency of crop plants under saline stress, which in turn contributes lower net assimilating and thus economic yield [9,18,20]. Moreover, various stress-management strategies at the cellular level—such as biosynthesis of antioxidants, active uptake of minerals, osmoregulation, and stress escape developments—cost an additional 10 times more energy compared to unstressed plants [10,75]. Thus, reprioritization of assimilates, adenosine triphosphate (ATP) and carbon partitioning result in lower grain/fruit yield in plants [7,9]. The results obtained from the current study have shown significant losses in grain yield and yield-related attributes, such as 100-grain weight, number of grains per ear, and grain weight per ear in maize plants under 120 mM NaCl (S3) compared to control (S1). However, the foliar application of EBL at 0.2 µM significantly improved grain yield and yield components compared to control/no EBL treatment (Table 1 and Table 4). Explicitly, EBL application in plants improves grain yield by alleviating saline and improving plant growth [31]. Rady [66] reported that exogenous application of EBL in *Phaseolus vulgaris* L. detoxified Na^+^-induced ionic stress and significantly improved yield and yield-contributing parameters. In pea plants, the EBL application increased the number of seeds and 1000-seed weight by up to 38% and 35%, respectively [76]. In short, the EBL application in plants can potentially improve yield by modulating a number of stress tolerance pathways, such as enhancing photosynthetic activity by stimulating biosynthesis of chlorophyll and improving enzymatic activity of rubisco and carbonic anhydrase [77], detoxification of ROS, and enhanced antioxidant activity [66] and gene upregulation [31,78]. However, the optimum dose, method of application, crop genetics, and stage of the crop in addition to the severity of the stress are the major aspects in deciding the relative increase in crop yield in response to exogenous EBL application. 

### 4.6. Correlations and PCA 

Moreover, Pearson’s correlation of the combinations of saline stress and exogenous EBL application showed a highly positive correlation between yield attributes and studied growth, physiological parameters, and chemical composition, which evidently supported the application of EBL as saline stress mitigator in maize. However, proline, SLW, K^+^, and antioxidant enzymatic activity depicted a negative relationship with yield attributes, which explicitly reflects their role in mitigating the deleterious effects of saline stress in response to EBL application [Figure 5]. Furthermore, the PCA (Figure 6) highlights the significant role of various parameters groups under the influence of saline stress and EBL application together. Thus, improving these traits through EBL application in crops may result in significant crop performance by enhancing saline stress tolerance. Similar multivariate analysis techniques, i.e., descriptive, ANOVA, PCA, and biplot analysis, were performed to determine its significance level under stress [79]. The results suggested that the phenotypic variation diminished across the observed factors and restricted diversity potential in the current study. A similar type of phenotypic variability across the observed factors and restricts the crop diversity was recorded in triticale [80] and fava bean [81] under stress conditions. Various effective multivariate strategies have been successfully utilized to identify tolerant and susceptible genotypes in diverse environmental conditions. Clustering analysis, regression approaches, and principal component analysis are widely used methodologies in plant breeding and screening programs [82]. The current study suggested that the samples treated with salinity and EBL forms cluster in one quadrant. Similar results were also reported by Otie et al. [82], suggesting that PCA results revealed that samples treated with saline and BRs formed clusters based on the frequencies of BRs’ applications.

## 5. Conclusions

In accordance with the results herein presented, the exogenous application of EBL efficiently alleviated saline stress in maize even under continuous 120 mM NaCl of salinity. The EBL treatments contribute to better plant growth, physiological traits, and higher photosynthetic capacity; facilitate antioxidant enzymatic activity; balance osmoregulation; and significant improve yield and yield attributes of maize under different saline stress intensities compared to untreated samples (EBL control). Even though saline stress deleteriously affects all studied parameters of maize plants, the exogenous application of 0.2 µM EBL significantly mitigated stress-mediated decline and enhanced plant tolerance against salinity. For instance, as compared to untreated plants (control), 0.2 µM EBL application improved biomass (+19%), rate of photosynthesis (+11%), and grain yield (+47%) of maize grown under salt stress. Thus, considering the EBL, a protective approach to alleviating saline stress in crop plants further exploring its mechanism of action, metabolic pathways, dose optimization, and crop specific response may lead us to an eco-friendly, economically viable, and efficient strategy to enhance agricultural productivity under saline environments.

## Figures and Tables

**Table 1:** Summary of analysis of variance (ANOVA) of all studies parameters for the effect of exogenous application of 24-Epibrassinolide (EBL) on maize plants grown under saline stress.

Parameters	Significance		
	Salinity	EBL	Salinity × EBL
Plant Height	**	**	NS
Stem Diameter	**	**	*
Biomass per Plant	**	**	NS
Leaf Area per Plant	**	**	**
Leaf Relative Water Contents (RWC)	**	**	*
Specific Leaf Weight (SLW)	**	**	**
Plant Growth Rate (PGR)	**	**	NS
Leaf Growth Rate (LGR)	**	**	**
Photochemical efficiency of PSII (Fv/Fm)	**	**	**
Leaf Green Index (LGI) (SPAD-Reading)	**	**	**
Rate of Photosynthesis (*Pn*)	**	**	**
Stomatal Conductance (Sc)	**	**	*
Transpiration Rate (TransR)	**	**	**
Intercellular CO_2_ Concentration (Ci)	**	**	NS
Intrinsic Water Use Efficiency (iWUE)	*	NS	NS
Carboxylation Efficiency (CE)	**	**	**
Proline	**	**	**
Total Phenolic Contents	**	**	*
Superoxide dismutase (SOD)	**	**	**
Catalase (CAT)	**	**	**
Ascorbate peroxidase (APX)	**	**	*
Na^+^ Concentration	**	**	**
K^+^ Concentration	**	**	**
Na^+^/K^+^	**	**	**
Number of Grains per Ear	**	**	NS
Grain Weight per Ear	**	**	NS
100-grain Weight	**	**	**
Grain Yield per Plant	**	**	**

** = *p* ≤ 0.01; * = *p* ≤ 0.05; NS = nonsignificant.

**Table 2:** The effect of exogenous application of 24-Epibrassinolide (EBL) on plant height, stem girth, biomass, leaf area per plant, leaf RWC, and SLW of maize plants grown under saline stress conditions.

Treatments/Parameters		Plant Height (cm)	Stem Diameter (mm)	Biomass (g/plant)	Leaf Area (cm^2^/plant)	Leaf RWC (%)	SLW (g/m^2^)
S1		228.56 A	18.32 A	114.94 A	6903 A	82.16 A	50.51 C
S2		213.67 B	17.13 B	99.60 B	5490 B	73.44 B	53.23 B
S3		187.11 C	16.10 C	86.98 C	4596 C	65.78 C	58.38 A
**LSD_0.05_**		**4.77**	**0.36**	**2.65**	**27.08**	**2.21**	**1.04**
**EBL**							
Control		191.78 C	15.79 C	92.96 C	4794 C	72.23 C	56.57 A
0.1 µM EBL		211.67 B	17.42 B	97.86 B	5725 B	73.58 B	53.61 B
0.2 µM EBL		225.89 A	18.34 A	110.71 A	6469 A	75.54 A	51.94 C
**LSD_0.05_**		**3.88**	**0.39**	**3.56**	**51.51**	**1.04**	**0.81**
**Salinity × EBL**							
**Salinity**	**EBL**						
S1	Control	209.67	16.37 de	108.20	6304 c	81.77 a	51.77 de
	0.1 µM EBL	230.67	18.93 b	114.83	6889 b	81.93 a	50.63 e
	0.2 µM EBL	245.33	19.67 a	121.80	7514 a	82.77 a	49.13 f
S2	Control	197.33	15.93 e	93.43	4726 g	71.73 c	55.27 c
	0.1 µM EBL	212.67	17.03 c	95.43	5587 f	73.13 c	52.97 d
	0.2 µM EBL	231.00	18.43 b	109.93	6158 d	75.47 b	51.47 e
S3	Control	168.33	15.07 f	77.23	3353 h	63.27 f	62.67 a
	0.1 µM EBL	191.67	16.30 de	93.30	4700 g	65.67 e	57.23 b
	0.2 µM EBL	201.33	16.93 cd	100.40	5734 e	68.40 d	55.23 c
**LSD_0.05_**		**NS**	**0.68**	**NS**	**89.22**	**1.80**	**1.40**

RWC = relative water contents; SLW = specific leaf weight; S1 = 0 mM NaCl, S2 = 60 mM NaCl, S3 = 120 mM NaCl; LSD = least significant differences. Capital letters show the significance differences between salt stress and EBL treatments, while small letters show the significance differences between the interaction effects of S × EBL.

**Table 3:** The effect of exogenous application of 24-Epibrassinolide (EBL) on leaf proline contents, total phenolic contents, and antioxidant enzymatic activity of maize plants grown under saline stress conditions.

Treatments/Parameters		Proline (mg/gFW)	Phenolic (mgGAE/gFM)	SOD (U/gFW)	CAT (U/gFW/min)	APX (umol/gFW/min)
**Salinity**						
S1		0.48 C	45.07 B	96.33 C	30.11 C	19.14 C
S2		1.91 B	56.08 A	134.44 B	47.23 B	28.11 B
S3		2.68 A	31.30 C	174.56 A	62.11 A	35.56 A
**LSD_0.05_**		**0.13**	**1.49**	**2.80**	**2.54**	**1.10**
**EBL**						
Control		2.38 A	41.06 C	126.56 C	42.56 C	25.33 C
0.1 µM EBL		1.60 B	44.43 B	133.89 B	46.56 B	27.11 B
0.2 µM EBL		1.09 C	47.00 A	144.89 A	50.22 A	30.33 A
**LSD_0.05_**		**0.53**	**1.73**	**2.26**	**0.97**	**1.41**
**Salinity × EBL**						
**Salinity**	**EBL**					
S1	Control	0.49 f	44.32 d	93.33 h	29.33 g	18.67 e
	0.1 µM EBL	0.49 f	45.76 d	95.67 h	30.00 g	18.33 e
	0.2 µM EBL	0.48 f	45.14 d	100.00 g	31.00 g	20.33 e
S2	Control	2.83 b	52.06 c	126.33 f	41.67 f	25.67 d
	0.1 µM EBL	1.86 d	56.06 b	134.33 e	47.33 e	27.67 d
	0.2 µM EBL	1.05 e	60.12 a	142.67 d	52.33 d	31.00 c
S3	Control	3.83 a	26.79 g	160.00 c	56.67 c	31.67 c
	0.1 µM EBL	2.45 c	31.47 f	171.67 b	62.33 b	35.33 b
	0.2 µM EBL	1.76 d	35.63 e	192.00 a	67.33 a	39.67 a
**LSD_0.05_**		**0.09**	**3.00**	**3.94**	**1.68**	**2.44**

SOD = superoxide dismutase; CAT = catalase; APX = ascorbate peroxidase; S1 = 0 mM NaCl, S2 = 60 mM NaCl, S3 = 120 mM NaCl; LSD = least significant differences. Capital letters show the significance differences between salt stress and EBL treatments, while small letters show the significance differences between the interaction effects of S × EBL.

**Table 4:** The effect of exogenous application of 24-Epibrassinolide (EBL) on number of grains per ear, grain weight per ear, 100-grain weight, and grain yield per plant of maize plants grown under saline stress conditions.

Treatments/Parameters		Number of Grains per Ear	Grain Weight per Ear (g)	100-grain Weight (g)	Grain Yield (g/plant)
**Salinity**					
S1		210.19 A	58.27 A	27.78 A	175.99 A
S2		161.02 B	38.51 B	23.98 B	101.00 B
S3		97.89 C	18.23 C	18.22 C	48.10 C
**LSD_0.05_**		**4.78**	**1.67**	**0.64**	**6.98**
**EBL**					
Control		136.67 C	32.02 C	22.07 C	88.16 C
0.1 µM EBL		153.44 B	37.29 B	23.33 B	107.67 B
0.2 µM EBL		178.78 A	45.70 A	24.58 A	129.24 A
**LSD_0.05_**		**5.81**	**1.89**	**0.27**	**4.14**
**Salinity × EBL**					
**Salinity**	**EBL**				
S1	Control	190.00	51.10	27.20 b	153.70 c
	0.1 µM EBL	204.05	56.33	27.60 b	170.30 b
	0.2 µM EBL	236.00	67.38	28.53 a	203.97 a
S2	Control	143.00	32.17	22.37 e	93.70 f
	0.1 µM EBL	163.03	38.43	24.20 d	101.47 e
	0.2 µM EBL	177.00	44.93	25.37 c	117.83 d
S3	Control	77.00	12.80	16.63 h	27.07 i
	0.1 µM EBL	93.33	17.10	18.20 g	51.23 h
	0.2 µM EBL	123.33	24.80	19.83 f	66.00 g
**LSD_0.05_**		**NS**	**NS**	**0.47**	**7.17**

S1 = 0 mM NaCl, S2 = 60 mM NaCl, S3 = 120 mM NaCl; LSD = least significant differences. Capital letters show the significance differences between salt stress and EBL treatments, while small letters show the significance differences among the interaction effects of S × EBL.

## Data Availability

The data are contained within the article.

## References

[1] Haj-Amor Z., Araya T., Kim D.G., Bouri S., Lee J., Ghiloufi W., Yang Y., Kang H., Jhariya M.K., Banerjee A. (2022). Soil salinity and its associated effects on soil microorganisms, greenhouse gas emissions, crop yield, biodiversity and desertification: A review. Sci. Total Environ..

[2] Taha R.S., Seleiman M.F., Alhammad B.A., Alkahtani J., Alwahibi M.S., Mahdi A.H. (2021). Activated Yeast extract enhances growth, anatomical structure, and productivity of *Lupinus termis* L. plants under actual salinity conditions. Agronomy.

[3] Dustgeer Z., Seleiman M.F., Khan I., Chattha M.U., Ali E.F., Alhammad B.A., Jalal R.S., Refay Y., Hassan M.U. (2021). Glycine-betaine induced salinity tolerance in maize by regulating the physiological attributes, antioxidant defense system and ionic homeostasis. Not. Bot. Horti Agrobot. Cluj-Napoca.

[4] Singh A. (2018). Alternative management options for irrigation-induced salinization and waterlogging under different climatic conditions. Ecol. Indic..

[5] Tian F., Hou M., Qiu Y., Zhang T., Yuan Y. (2020). Salinity stress effects on transpiration and plant growth under different salinity soil levels based on thermal infrared remote (TIR) technique. Geoderma.

[6] Hopmans J.W., Qureshi A.S., Kisekka I., Munns R., Grattan S.R., Rengasamy P., Ben-Gal A., Assouline S., Javaux M., Minhas P.S. (2021). Critical knowledge gaps and research priorities in global soil salinity. Adv. Agron..

[7] Kumawat K.C., Nagpal S., Sharma P. (2022). Potential of plant growth-promoting rhizobacteria-plant interactions in mitigating salt stress for sustainable agriculture: A review. Pedosphere.

[8] Lodeyro A.F., Carrillo N. (2015). Salt stress in higher plants: Mechanisms of toxicity and defensive responses. Salt Stress in Higher Plants: Mechanisms of Toxicity and Defensive Responses.

[9] Alhammad B.A., Ahmad A., Seleiman M.F., Tola E. (2023). Seed priming with nanoparticles and 24-epibrassinolide improved seed germination and enzymatic performance of *Zea mays* L. in salt-stressed soil. Plants.

[10] Alkharabsheh H.M., Seleiman M.F., Hewedy O.A., Battaglia M.L., Jalal R.S., Alhammad B.A., Schillaci C., Ali N., Al-Doss A. (2021). Field crop responses and management strategies to mitigate soil salinity in modern agriculture: A review. Agronomy.

[11] Seleiman M.F., Al-Suhaibani N., Ali N., Akmal M., Alotaibi M., Refay Y., Dindaroglu T., Abdul-Wajid H.H., Battaglia M.L. (2021). Drought stress impacts on plants and different approaches to alleviate its adverse effects. Plants.

[12] Zhu J.K. (2016). Abiotic stress signaling and responses in plants. Cell.

[13] Seleiman M.F., Semida W.M., Rady M.M., Mohamed G.F., Hemida K.A., Alhammad B.A., Hassan M.M., Shami A. (2020). Sequential application of antioxidants rectifies ion imbalance and strengthens antioxidant systems in salt-stressed cucumber. Plants.

[14] Ahanger M.A., Tomar N.S., Tittal M., Argal S., Agarwal R. (2017). Plant growth under water/salt stress: ROS production; antioxidants and significance of added potassium under such conditions. Physiol. Mol. Biol. Plants.

[15] Singh A., Roychoudhury A. (2021). Gene regulation at transcriptional and post-transcriptional levels to combat salt stress in plants. Physiol. Plant..

[16] Qin H., Huang R. (2020). The phytohormonal regulation of Na^+^/K^+^ and reactive oxygen species homeostasis in rice salt response. Mol. Breed..

[17] Zhao C., Zhang H., Song C., Zhu J.K., Shabala S. (2020). Mechanisms of plant responses and adaptation to soil salinity. Innovation.

[18] Wegner L.H., Stefano G., Shabala L., Rossi M., Mancuso S., Shabala S. (2011). Sequential depolarization of root cortical and stelar cells induced by an acute salt shock–implications for Na^+^ and K^+^ transport into xylem vessels. Plant Cell Environ..

[19] Yang Y., Guo Y. (2018). Unraveling salt stress signaling in plants. J. Integr. Plant Biol..

[20] Yang Y., Guo Y. (2018). Elucidating the molecular mechanisms mediating plant salt-stress responses. New Phytol..

[21] Shabala S., Pottosin I. (2014). Regulation of potassium transport in plants under hostile conditions: Implications for abiotic and biotic stress tolerance. Physiol. Plant..

[22] Munns R., James R.A., Gilliham M., Flowers T.J., Colmer T.D. (2016). Tissue tolerance: An essential but elusive trait for salt-tolerant crops. Funct. Plant Biol..

[23] Razzaq A., Ali A., Safdar L.B., Zafar M.M., Rui Y., Shakeel A., Shaukat A., Ashraf M., Gong W., Yuan Y. (2020). Salt stress induces physiochemical alterations in rice grain composition and quality. J. Food Sci..

[24] Awais A., El-Kamil T., Thobayet S., Alshahrani S., Seleiman M.F. (2023). Enhancement of morphological and physiological performance of *Zea mays* L. under saline stress using ZnO nanoparticles and 24-epibrassinolide seed priming. Agronomy.

[25] Seleiman M.F., Ahmad A., Alshahrani T.S. (2023). Integrative effects of zinc nanoparticle and PGRs to mitigate salt stress in maize. Agronomy.

[26] Shahbaz M., Ashraf M., Athar H.U.R. (2008). Does exogenous application of 24-epibrassinolide ameliorate salt induced growth inhibition in wheat (*Triticum aestivum* L.)?. Plant Growth Regul..

[27] Tanveer M. (2019). Role of 24-Epibrassinolide in inducing thermo-tolerance in plants. J. Plant Growth Regul..

[28] Siddiqui H., Ahmed K.B.M., Hayat S. (2018). Comparative effect of 28-homobrassinolide and 24-epibrassinolide on the performance of different components influencing the photosynthetic machinery in *Brassica juncea* L. Plant Physiol. Biochem..

[29] Tanveer M., Shahzad B., Sharma A., Khan E.A. (2019). 24-Epibrassinolide application in plants: An implication for improving drought stress tolerance in plants. Plant Physiol. Biochem..

[30] Shahzad B., Tanveer M., Che Z., Rehman A., Cheema S.A., Sharma A., Song H., ur Rehman S., Zhaorong D. (2018). Role of 24-epibrassinolide (EBL) in mediating heavy metal and pesticide induced oxidative stress in plants: A review. Ecotoxicol. Environ. Saf..

[31] Tanveer M., Shahzad B., Sharma A., Biju S., Bhardwaj R. (2018). 24-Epibrassinolide; an active brassinolide and its role in salt stress tolerance in plants: A review. Plant Physiol. Biochem..

[32] Anastassiadou M., Arena M., Auteri D., Brancato A., Bura L., Carrasco Cabrera L., Chaideftou E., Chiusolo A., Marques D.C., European Food Safety Authority (EFSA) (2020). Peer review of the pesticide risk assessment of the active substance 24-epibrassinolide. EFSA J..

[33] Seleiman M.F., Selim S., Jaakkola S., Mäkelä P.S. (2017). Chemical composition and in vitro digestibility of whole-crop maize fertilized with synthetic fertilizer or digestate and harvested at two maturity stages in Boreal growing conditions. Agric. Food Sci..

[34] Reynolds T.W., Waddington S.R., Anderson C.L., Chew A., True Z., Cullen A. (2015). Environmental impacts and constraints associated with the production of major food crops in Sub-Saharan Africa and South Asia. Food Secur..

[35] Erenstein O., Chamberlin J., Sonder K. (2021). Estimating the global number and distribution of maize and wheat farms. Glob. Food Secur..

[36] Grote U., Fasse A., Nguyen T.T., Erenstein O. (2021). Food security and the dynamics of wheat and maize value chains in Africa and Asia. Front. Sustain. Food Syst..

[37] Wang J., Hu X. (2021). Research on corn production efficiency and influencing factors of typical farms: Based on data from 12 corn-producing countries from 2012 to 2019. PLoS ONE.

[38] Basit F., Chen M., Ahmed T., Shahid M., Noman M., Liu J., An J., Hashem A., Fahad Al-Arjani A.B., Alqarawi A.A. (2021). Seed priming with brassinosteroids alleviates chromium stress in rice cultivars via improving ROS metabolism and antioxidant defense response at biochemical and molecular levels. Antioxidants.

[39] Wolf B. (1982). The comprehensive system of leaf analysis and its use for diagnosing crop nutrient status. Commun. Soil Sci. Plant Anal..

[40] Jiang M., Zhang J. (2002). Water stress-induced abscisic acid accumulation triggers the increased generation of reactive oxygen species and up-regulates the activities of antioxidant enzymes in maize leaves. J. Exp. Bot..

[41] Zhu Z., Wei G., Li J., Qian Q., Yu J. (2004). Silicon alleviates salt stress and increases antioxidant enzymes activity in leaves of salt-stressed cucumber (*Cucumis sativus* L.). Plant Sci..

[42] Kong L., Wang M., Bi D. (2005). Selenium modulates the activities of antioxidant enzymes, osmotic homeostasis and promotes the growth of sorrel seedlings under salt stress. Plant Growth Regul..

[43] Tawaha K., Alali F.Q., Gharaibeh M., Mohammad M., El-Elimat T. (2017). Antioxidant activity and total phenolic content of selected Jordanian plant species. Food Chem..

[44] Singleton V.L., Rossi J.A. (1965). Colorimetry of total phenolics with phosphomolybdic-phosphotungstic acid reagents. Am. J. Enol. Vitic..

[45] Bates L.S., Waldren R.A., Teare I.D. (1973). Rapid determination of free proline for water-stress studies. Plant Soil.

[46] Farooq M., Hussain M., Wakeel A., Siddique K.H. (2015). Salt stress in maize: Effects, resistance mechanisms, and management. A review. Agron. Sustain. Dev..

[47] Jiang C., Zu C., Lu D., Zheng Q., Shen J., Wang H., Li D. (2017). Effect of exogenous selenium supply on photosynthesis, Na^+^ accumulation and antioxidative capacity of maize (*Zea mays* L.) under salinity stress. Sci. Rep..

[48] Talat N. (2020). Alleviation of soil salinization and the management of saline soils, climate change, and soil interactions. Climate Change and Soil Interactions.

[49] Almeida D.M., Oliveira M.M., Saibo N.J. (2017). Regulation of Na^+^ and K^+^ homeostasis in plants: Towards improved salt stress tolerance in crop plants. Genet. Mol. Biol..

[50] Guo Q., Liu L., Barkla B.J. (2019). Membrane lipid remodeling in response to salinity. Int. J. Mol. Sci..

[51] Ahmad P., Ahanger M.A., Egamberdieva D., Alam P., Alyemeni M.N., Ashraf M. (2018). Modification of osmolytes and antioxidant enzymes by 24-epibrassinolide in chickpea seedlings under mercury (Hg) toxicity. J. Plant Growth Regul..

[52] Desoky E.S.M., Mansour E., Ali M.M., Yasin M.A., Abdul-Hamid M.I., Rady M.M., Ali E.F. (2021). Exogenously used 24-epibrassinolide promotes drought tolerance in maize hybrids by improving plant and water productivity in an arid environment. Plants.

[53] Nolan T.M., Vukašinović N., Liu D., Russinova E., Yin Y. (2020). Brassinosteroids: Multidimensional regulators of plant growth, development, and stress responses. Plant Cell.

[54] Khan I., Awan S.A., Ikram R., Rizwan M., Akhtar N., Yasmin H., Sayyed R.Z., Ali S., Ilyas N. (2021). Effects of 24-epibrassinolide on plant growth, antioxidants defense system, and endogenous hormones in two wheat varieties under drought stress. Physiol. Plant..

[55] Shahzad R., Harlina P.W., Ewas M., Zhenyuan P., Nie X., Gallego P.P., Ullah Khan S., Nishawy E., Khan A.H., Jia H. (2021). Foliar applied 24-epibrassinolide alleviates salt stress in rice (*Oryza sativa* L.) by suppression of ABA levels and upregulation of secondary metabolites. J. Plant Interact..

[56] Yue J., Fu Z., Zhang L., Zhang Z., Zhang J. (2018). The positive effect of different 24-epiBL pretreatments on salinity tolerance in *Robinia pseudoacacia* L. seedlings. Forests.

[57] Yang Z., Li J.L., Liu L.N., Xie Q., Sui N. (2020). Photosynthetic regulation under salt stress and salt-tolerance mechanism of sweet sorghum. Front. Plant Sci..

[58] Shin Y.K., Bhandari S.R., Jo J.S., Song J.W., Cho M.C., Yang E.Y., Lee J.G. (2020). Response to salt stress in lettuce: Changes in chlorophyll fluorescence parameters, phytochemical contents, and antioxidant activities. Agronomy.

[59] Acosta-Motos J.R., Ortuño M.F., Bernal-Vicente A., Diaz-Vivancos P., Sanchez-Blanco M.J., Hernandez J.A. (2017). Plant responses to salt stress: Adaptive mechanisms. Agronomy.

[60] Yun P., Xu L., Wang S.S., Shabala L., Shabala S., Zhang W.Y. (2018). *Piriformospora indica* improves salinity stress tolerance in *Zea mays* L. plants by regulating Na^+^ and K^+^ loading in root and allocating K^+^ in shoot. Plant Growth Regul..

[61] Munns R., Tester M. (2008). Mechanisms of salinity tolerance. Annu. Rev. Plant Biol..

[62] Babitha K.C., Vemanna R.S., Nataraja K.N., Udayakumar M. (2015). Overexpression of EcbHLH57 transcription factor from *Eleusine coracana* L. in tobacco confers tolerance to salt, oxidative and drought stress. PLoS ONE.

[63] Mittler R. (2002). Oxidative stress, antioxidants and stress tolerance. Trends Plant Sci..

[64] Van Raamsdonk J.M., Hekimi S. (2012). Superoxide dismutase is dispensable for normal animal lifespan. Proc. Natl. Acad. Sci. USA.

[65] Sharma I., Pati P.K., Bhardwaj R. (2011). Effect of 24-epibrassinolide on oxidative stress markers induced by nickel-ion in *Raphanus sativus* L. Acta Physiol. Plant..

[66] Rady M.M. (2011). Effect of 24-epibrassinolide on growth, yield, antioxidant system and cadmium content of bean (*Phaseolus vulgaris* L.) plants under salinity and cadmium stress. Sci. Hortic..

[67] Agami R.A. (2013). Alleviating the adverse effects of NaCl stress in maize seedlings by pretreating seeds with salicylic acid and 24-epibrassinolide. S. Afr. J. Bot..

[68] Goda H., Shimada Y., Asami T., Fujioka S., Yoshida S. (2002). Microarray analysis of brassinosteroid-regulated genes in *Arabidopsis*. Plant Physiol..

[69] Yuan L., Shu S., Sun J., Guo S., Tezuka T. (2012). Effects of 24-epibrassinolide on the photosynthetic characteristics, antioxidant system, and chloroplast ultrastructure in *Cucumis sativus* L. under Ca(NO_3_)_2_ stress. Photosynth. Res..

[70] Zhu J.K. (2003). Regulation of ion homeostasis under salt stress. Curr. Opin. Plant Biol..

[71] Abdelaziz M.E., Kim D., Ali S., Fedoroff N.V., Al-Babili S. (2017). The endophytic fungus *Piriformospora indica* enhances *Arabidopsis thaliana* growth and modulates Na^+^/K^+^ homeostasis under salt stress conditions. Plant Sci..

[72] Dong Y., Wang W., Hu G., Chen W., Zhuge Y., Wang Z., He M.R. (2017). Role of exogenous 24-epibrassinolide in enhancing the salt tolerance of wheat seedlings. J. Soil Sci. Plant Nutr..

[73] Azhar N., Su N., Shabala L., Shabala S. (2017). Exogenously applied 24-epibrassinolide (EBL) ameliorates detrimental effects of salinity by reducing K^+^ efflux via depolarization-activated K^+^ channels. Plant Cell Physiol..

[74] Das P., Nutan K.K., Singla-Pareek S.L., Pareek A. (2015). Understanding salinity responses and adopting ‘omics-based’ approaches to generate salinity tolerant cultivars of rice. Front. Plant Sci..

[75] Shabala S., Shabala S., Cuin T.A., Pang J., Percey W., Chen Z., Conn S., Eing C., Wegner L.H. (2010). Xylem ionic relations and salinity tolerance in barley. Plant J..

[76] Shahid M., Naeem-Ullah U., Khan W., Saeed D.S., Razzaq K. (2021). Application of nanotechnology for insect pests management: A review. J. Innov. Sci..

[77] Yu J.Q., Huang L.F., Hu W.H., Zhou Y.H., Mao W.H., Ye S.F., Nogués S. (2004). A role for brassinosteroids in the regulation of photosynthesis in *Cucumis sativus*. J. Exp. Bot..

[78] Ali B., Hayat S., Ahmad A. (2007). 28-Homobrassinolide ameliorates the saline stress in chickpea (*Cicer arietinum* L.). Environ. Exp. Bot..

[79] Sneath P.H., Sokal R.R. (1973). Numerical Taxonomy. The Principles and Practice of Numerical Classification.

[80] Saed-Moucheshi A., Pessarakli M., Heidari B. (2013). Comparing relationships among yield and its related traits in mycorrhizal and nonmycorrhizal inoculated wheat cultivars under different water regimes using multivariate statistics. Int. J. Agron..

[81] Afzal M., Alghamdi S.S., Migdadi H.H., El-Harty E., Al-Faifi S.A. (2022). Agronomical and physiological responses of faba bean genotypes to salt stress. Agriculture.

[82] Otie V., Ibrahim A., Udo I., Kashiwagi J., Matsuura A., Shao Y., Itam M., An P., Eneji A.E. (2022). Foliarly applied 24-Epibrassinolide modulates the electrical conductivity of the saturated rhizospheric soil extracts of soybean under salinity stress. Plants.

